# Dissociable Patterns of Atypical Error Monitoring in Developmental Dyslexia and Attention Deficit Hyperactivity Disorder

**DOI:** 10.3390/brainsci16070669

**Published:** 2026-06-26

**Authors:** Xueqing Wang, Jiuju Wang, Jiaqi Cao, Zhifang Wang, Jing Zhao

**Affiliations:** 1 School of Psychology, Capital Normal University, Beijing 100048, China; 2 Institute of Mental Health, Peking University Sixth Hospital, Beijing 100083, China; 3 NHC Key Laboratory of Mental Health, Peking University, Beijing 100871, China; 4 National Clinical Research Center for Mental Disorders, Peking University Sixth Hospital, Beijing 100083, China

**Keywords:** dyslexia, attention deficit hyperactivity disorder, comorbidity, post error, error monitoring

## Abstract

**Highlights:**

**What are the main findings?**

- Dissociable alterations in error monitoring were observed, that is, dyslexia was associated with atypical error detection indexed by reduced post-error slowing, while ADHD was linked to less efficient error regulation indexed by lower post-error accuracy.
- Post-error slowing independently predicted character recognition performance but not ADHD symptom severity.

**What are the implications of the main findings?**

- Weak error detection and altered error regulation may represent distinct cognitive profiles associated with developmental dyslexia and ADHD, respectively.
- These findings suggest a potential mechanism for the comorbidity of dyslexia and ADHD, providing tentative implications for intervention strategies.

**Abstract:**

This study investigated whether alterations in error monitoring constitute shared risk factors for developmental dyslexia (DD) and attention deficit hyperactivity disorder (ADHD). A total of 148 children were recruited and categorized into four groups, including 37 children with DD only, 37 with ADHD only, 40 with comorbid DD + ADHD, and 34 typically developing (TD) controls. Participants completed a combined Flanker–No-Go task to assess error monitoring, utilizing post-error slowing (PES) and post-error accuracy (PEA) to index error detection and error regulation, respectively. Results of linear mixed-effects models showed a significant main effect of DD_status on PES, indicating that children with DD (including the DD-only and comorbid DD + ADHD groups) exhibited significantly shorter PES than their non-DD counterparts. Conversely, a significant main effect of ADHD_status was observed on PEA, where children with ADHD showed lower PEA relative to those without ADHD. Notably, there were no significant differences in error monitoring functions between children with single disorders and those with comorbidities. Furthermore, hierarchical regression analyses demonstrated that PES independently predicted character recognition, but not ADHD symptom severity. These findings suggest that while both DD and ADHD involve atypical error monitoring, the underlying patterns are dissociable. Specifically, the DD profile tended to align with altered error detection, whereas the ADHD profile was more closely associated with reduced error regulation. Crucially, the comorbid group exhibited a combination of the error monitoring problems observed in DD and ADHD.

## 1. Introduction

Developmental dyslexia (DD) and attention deficit hyperactivity disorder (ADHD) are two prevalent neurodevelopmental disorders in children and adolescents [1,2]. As a specific learning disorder, DD is characterized by persistent difficulties in word recognition, reading fluency, and/or spelling ability, even after excluding factors such as intellectual disability, inadequate educational opportunities, and sensory impairments [3]. Its prevalence in the general population is approximately 4.9–6.9% [4]. ADHD is primarily defined by core symptoms of inattention, disorganization, and/or hyperactivity–impulsivity. Specifically, inattention symptoms manifest as an inability to sustain focus, apparent distractibility, and disorganization of materials, while hyperactivity–impulsivity involves excessive motor activity, fidgeting, intruding on others, and an inability to wait. These symptoms are inconsistent with the individual’s developmental level [5]. The prevalence of ADHD is estimated at 6.4–10.5% [6].

Importantly, DD and ADHD frequently co-occur, with a comorbidity rate of 25–48%, much higher than chance [7]. Compared to children with a single disorder, those with comorbid DD and ADHD face more severe challenges in academic achievement, social adaptation, and occupational functioning, including higher rates of grade retention, behavioral problems, and legal risks [8]. Consequently, it is important to elucidate the cognitive profile of children with comorbid DD and ADHD for developing targeted intervention strategies. To explain such comorbidity, one of the main hypotheses, the multiple-deficit model [9,10], posits that comorbidity arises, at least in part, from shared etiological factors in the genetic, neurobiological, and cognitive levels between disorders. Within this framework, identifying shared cognitive deficits is essential.

One potential shared deficit is impaired error monitoring. Children with DD and ADHD frequently failed to detect or adjust to their own mistakes during cognitive tasks and daily activities [11,12]. For instance, they may overlook misreadings during oral reading or ignore execution errors in routine tasks. Consequently, determining the role of error monitoring as a core shared cognitive vulnerability is critical for advancing our understanding of the co-occurrence between DD and ADHD. Error monitoring refers to the ability to promptly detect and initiate corrective responses to errors. It represents a core metacognitive function and an essential component of executive control [13], playing a pivotal role in behavioral regulation and adaptive learning [14,15,16]. This process generally comprises two subcomponents: error detection and error regulation. Error detection involves the timely identification of a mistake, which provides the necessary feedback for subsequent correction. In contrast, error regulation entails the strategic adjustment of behavior to prevent error recurrence [17].

Early studies frequently assessed error monitoring using the error awareness task, quantifying the error awareness rate as the proportion of errors explicitly reported relative to the total number of errors committed [18]. Research indicates that both children with DD and those with ADHD exhibit significantly lower error awareness rates compared to age-matched typically developing peers [18,19], suggesting that the error monitoring deficit may be a shared characteristic across these disorders. However, reliance on subjective reports and a predominant focus on error detection limit the ability of such tasks to comprehensively and objectively capture the full scope of error monitoring, particularly the dimension of error regulation.

To overcome the limitations of self-reporting, subsequent studies employed the antisaccade task with eye-tracking to provide a more objective and comprehensive assessment of error detection and regulation functions. In this paradigm, participants are required to suppress the prepotent urge to look at a suddenly appearing peripheral target and instead execute a saccade to the opposite location. Researchers typically quantify error monitoring ability using the saccade directional error rate (i.e., the proportion of trials with an initial erroneous saccade toward the target), correction rate (i.e., the proportion of initial errors successfully rectified), and omission rate (i.e., the proportion of uncorrected initial errors) [19,20,21,22,23,24,25]. While studies consistently report higher directional error rates in both clinical groups [20,21,22,26,27], the overall error rate is confounded by response inhibition. In contrast, correction and omission rates are more specific to error monitoring. Previous studies using these specific indicators have primarily centered on the DD population. Findings indicate that, compared to age-matched typically developing children, children with DD exhibit a lower correction rate and a higher omission rate in the antisaccade task [20,21,22], pointing to distinct impairments in error monitoring. To further investigate the cognitive mechanisms underlying error monitoring, Zhai et al. [19] designed a combined antisaccade and error-awareness task, requiring participants to subjectively report whether they had fixated on the target after a reverse saccade, thereby distinguishing between conscious and unconscious errors. Their results revealed that group differences were significant only in the unconscious error condition; in the conscious error condition, children with DD did not differ significantly from those of age-matched typically developing children. These findings suggest that the poorer antisaccade performance in children with DD may primarily stem from difficulties in error detection, whereas their ability to correct detected errors remains relatively intact. Nevertheless, antisaccade performance is inherently reliant on oculomotor control, and deficits in this domain have been consistently documented in both DD and ADHD populations [28,29]. Therefore, impaired antisaccade performance could be attributed to either specific deficits in error monitoring or limitations in basic oculomotor control.

Given these potential confounds, researchers have increasingly turned to button-press tasks, such as the Go/No-Go and Flanker tasks, using post-error effects as indirect indices. Upon detecting an error, individuals typically decelerate their subsequent response to prioritize accuracy. This phenomenon, known as post-error slowing (PES), is widely regarded as a behavioral indicator of error detection [30]. Generally, a more pronounced PES indicates a more robust error detection function. Furthermore, post-error accuracy (PEA), defined as the accuracy of the response in the trial immediately following an error trial, reflects error regulation [30], with higher PEA signifying a stronger ability to correct errors. 

Previous studies employing these measures have predominantly focused on individuals with ADHD, but the findings remain controversial. Some studies have found that, compared to age-matched typically developing children, children with ADHD exhibit shorter PES [15,30,31,32] and lower PEA [33] across the stop-signal, Go/No-Go, and Flanker tasks. These results suggest concurrent deficits in both error detection and error regulation. However, other studies have failed to replicate these findings [30,34]. For instance, Arnett et al. [30] and van Meel et al. [34] found no significant group differences in either PEA or PES between children with ADHD and the controls using the stop-signal and Flanker tasks, respectively. To address these inconsistencies, Ding and Yang [35] proposed an alternative interpretation. By incorporating subjective reports of error awareness into a Go/No-Go task to prompt explicit error noticing, they found that children with ADHD exhibited a level of PES comparable to that of their typically developing peers. The authors concluded that the error detection function in individuals with ADHD is not inherently impaired; rather, the previously observed attenuation of PES may stem from the insufficient time allocated for error identification. Nevertheless, the methodological limitations warrant consideration. The relatively low cognitive load of the task may reduce the demand on the higher-order function of error monitoring, thereby masking potential group differences and failing to fully capture the deficit profile of children with ADHD. Consequently, due to variations in task paradigms and potential methodological constraints, the pattern of post-error effects in children with ADHD remains to be further clarified.

Based on the above, the present study employed a combined Flanker–No-Go task, with dual behavioral indicators (i.e., PES and PEA) to systematically compare the error monitoring functions across three clinical groups including children with DD, those with ADHD, those with comorbid conditions, and a control group of age-matched typically developing children. According to the previous literature [19,20,21,22,23,30,31,32,33], we hypothesize that children with DD may exhibit shorter PES compared to typically developing children, reflecting atypical error detection, and children with ADHD may show both shorter PES and lower PEA than their TD peers, reflecting alterations in both error detection and regulation; meanwhile, children with comorbid conditions might demonstrate a cumulative effect of the cognitive alterations associated with both disorders. These patterns would suggest shared problems in error detection between DD and ADHD, alongside a relatively specific weakness in error regulation for ADHD.

## 2. Methods

### 2.1. Participants

A total of 148 children participated in this study, comprising 34 children in the typically developing (TD) group (22 males, 12 females; mean age = 7.93 ± 0.52 years), 37 children in the DD-only group (22 males, 15 females; mean age = 8.05 ± 0.69 years), 37 children in the ADHD-only group (31 males, 6 females; mean age = 9.98 ± 1.28 years), and 40 children in the comorbid DD + ADHD group (31 males, 9 females; mean age = 10.02 ± 1.63 years). Post-hoc power analysis using G*Power 3.1 indicated that the current sample achieved a statistical power of approximately 0.88 to detect a medium effect size. Participants were recruited from partner elementary schools, recommendations from hospitals, and online advertisements. Detailed descriptive statistics for each group are presented in Table 1.

**Screening criteria for ADHD**. Participants were classified as having ADHD if they met the following criteria: (1) receiving a positive diagnosis based on the Schedule for Affective Disorders and Schizophrenia for School-Age Children-Present and Lifetime Version (K-SADS-PL) administered by child psychiatrists; (2) meeting at least 6 items on either the inattentive or hyperactive/impulsive subscales of the parent-reported Swanson, Nolan, and Pelham Rating Scale IV (SNAP-IV).

**Screening criteria for DD.** For children recruited through hospitals and online advertisements, dyslexia was screened using the Chinese character recognition test and the word-list reading fluency test [36,37]. Children were classified as having DD if they met either of the following criteria: (1) scoring below at least −1.5 standard deviations of the grade-level mean on the Chinese character recognition test; or (2) scoring below at least −1 standard deviation of the grade-level mean on the Chinese character recognition test and below at least −1.5 standard deviations of the grade-level mean on the word-list reading fluency test. For children recruited from elementary schools, dyslexia was identified using the character recognition measure and assessment scale for primary school children [38]. Children scoring below −1.5 standard deviations of the age-level average and identified by their teachers as having difficulties in Chinese language learning were classified as having DD.

Children meeting only the ADHD screening criteria were assigned to the ADHD-only group; those meeting only the DD screening criteria were enrolled in the DD-only group; and those meeting both the ADHD and DD screening criteria were assigned to the comorbid group.

Inclusion criteria for the TD group: These included scoring fewer than four positive items on both the inattention subscale and the hyperactivity–impulsivity subscale of the SNAP-IV, and demonstrating average or above-average performance on the aforementioned dyslexia screening measures.

Exclusion criteria for all groups: These included scoring below the 25th percentile on the Raven’s standard progressive matrices test (RSPM) [39], and the presence of primary emotional disorders (e.g., depression or anxiety) or psychiatric disorders (e.g., schizophrenia). It should be noted that this study did not collect data regarding medication status, particularly for children with ADHD symptoms (i.e., the ADHD-only and DD + ADHD comorbidity groups). Given that the current cohort of children with ADHD were predominantly recruited from clinical hospitals, a proportion of these children were likely receiving pharmacological treatment. Since stimulant medications are known to enhance error-monitoring functions [40], we acknowledge that medication effects may have potentially confounded task performance in the error monitoring task in our ADHD sample.

This study was approved by the Ethics Review Committee of School of Psychology, Capital Normal University. All procedures adhered to the ethical standards outlined in the *Declaration of Helsinki*. Written informed consent was obtained from the parents of all participants before the experiment.

### 2.2. Screening Tools

#### 2.2.1. A Screening Test for Attention Deficit Hyperactivity Disorder

The parent-reported Swanson, Nolan, and Pelham Rating Scale IV [41] was used in this study. The scale comprises 18 items rated on a four-point Likert scale ranging from 0 (never) to 3 (always). The first nine items constitute the inattentive subscale, and the remaining nine items constitute the hyperactive–impulsive subscale. In this study, items rated at 2 and 3 points were classified as positive. The number of positive items was calculated to assess the presence of ADHD, while the total scores in each subscale were used as statistical indicators for inattentive and hyperactive–impulsive symptoms, respectively. According to Chinese normative data, the test–retest reliability of this scale is 0.72, with a specificity of 0.90 and a sensitivity of 0.92 for diagnosing ADHD.

#### 2.2.2. A Screening Test for Dyslexia

**Chinese character recognition test** [37]. This individually administered test was used to assess participants’ character recognition ability. Participants were required to read aloud 150 Chinese characters. The number of characters read correctly was recorded as the raw score, which was converted into a standardized score based on an established norm [37]. This test has demonstrated high test–retest reliability, ranging from 0.84 to 0.97.

**Word-list reading fluency test** [36]. This test was administered individually to assess word-level reading fluency. Participants were required to read aloud 180 two-character words as quickly as possible. The number of words read correctly per minute were computed as the final score, serving as an indicator of the participants’ reading fluency. The test–retest reliability of this measure ranges from 0.83 to 0.97.

**Character recognition measure and assessment scale for primary school children** [38]. Due to time constraints imposed by the participating primary schools, this paper-to-pencil test we administered in a group setting to facilitate efficiency. Participants were required to demonstrate their recognition of target characters by providing the corresponding Pinyin or forming a compound word containing the target character. For example, for the character “学” (/xue2/, meaning study), a correct response would be writing a compound word such as “学习” (/xue2xi2/, meaning studying) or annotating it with Pinyin /xue2/. One point was awarded for each valid compound word or correct Pinyin annotation. The characters were divided into 10 groups according to ascending difficulty, with each group assigned a specific difficulty coefficient. The score for each group of characters was calculated by multiplying the total points by the corresponding coefficient of difficulty. The final score for each participant was the sum of the subscores for all 10 groups, which revealed the estimation of the participant’s vocabulary size, and further, reflecting their Chinese-character recognition ability. This scale has demonstrated high reliability and validity (both reported as 0.98). However, given that performances on this task may be potentially confounded by handwriting and word knowledge, these limitations will be explicitly addressed in the Section 4.

#### 2.2.3. Non-Verbal Intelligence Tests

The Chinese Revised Version of the Raven’s Standard Progressive Matrices [39] was used to assess non-verbal intelligence. The test consists of 60 items divided into five sets of 12 items in each set, with gradually increasing difficulty. Each item consists of a target figure with a missing part and 6–8 options. Participants were required to select the optimal option to complete the target figure. One point was awarded for each correct answer. The raw score was the number of correct responses, which was converted into the standardized score based on the Chinese norms established by Zhang and Wang [39]. The Cronbach’s alpha reliability for this test was 0.89.

### 2.3. Error Monitoring Task

A combined Flanker–No-Go paradigm [42,43] was used to assess participants’ error monitoring function. The task was programmed and administered online by a web-based tool named PsyToolkit (https://www.psytoolkit.org) [44,45], with stimuli displayed in white on a black background. The procedure consisted of a practice phase (16 trials) and a formal test phase (160 trials). The practice phase was used to familiarize participants with the task and materials. The formal test was conducted in either three or four blocks: the three-block version comprised 64, 48, and 48 trials, respectively; while the four-block version contained 40 trials per block. Specifically, nine children with comorbid DD and ADHD who were drawn from a hospital-based neuroimaging cohort received the three-block version. These participants completed the three-block version of the task immediately outside the scanner following their imaging session. This adjustment was necessary to align the behavioral assessment with the specific time-series requirements of the neuroimaging protocol. To verify whether this variation confounded our results or not, we compared the post-error monitoring indices between the comorbid children who completed the three-block version and those who completed the four-block version. No significant differences were found between the two subgroups, indicating that the task version did not impact task performance (see Table S1 in Supplementary Materials for detailed data).

**Figure 1:**
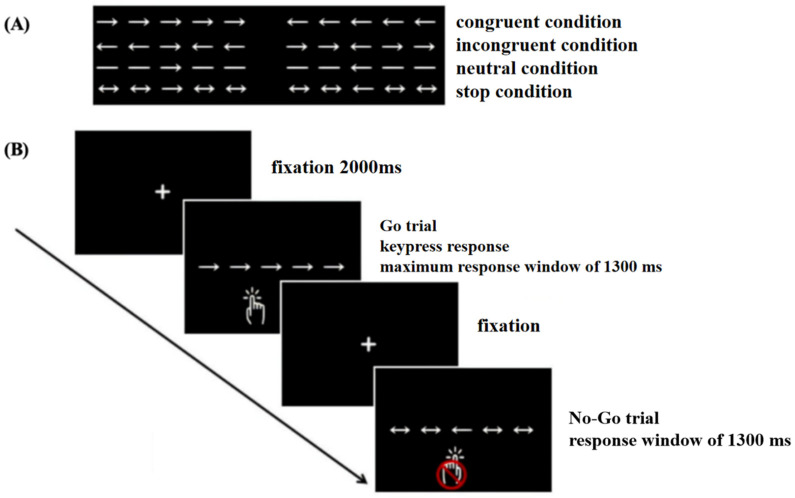
Stimulus material of the combined Flanker–No-Go task (**A**) and flowchart of the trial procedure (**B**). For panel (**A**), in the congruent condition, the central arrow pointed in the same direction as the flanking arrows; in incongruent condition, the central arrow pointed in the opposite direction to the flanking arrows; in the neutral condition, the flanking stimuli were line segments of equal length to the central arrow; in the stop condition, the flanking stimuli were bidirectional arrows. The congruent, incongruent, and neutral conditions were all designated as Go trials, whereas the stop condition corresponded to No-Go trials.

There were four conditions of the stimulus presentation, including congruent, incongruent, neutral, and stop conditions, each constituting 25% of the total trials. As shown in Figure 1A, the stimulus materials consisted of a sequence of five arrows or line segments. The four conditions were distinguished based on the relationship between the central arrow and the flanking arrows or segments: (1) congruent condition: the central arrow pointed in the same direction as the flanking arrows; (2) incongruent condition: the central arrow pointed in the opposite direction to the flanking arrows; (3) neutral condition: the flanking stimuli were line segments of equal length to the central arrow; (4) stop condition: the flanking stimuli were bidirectional arrows. The congruent, incongruent, and neutral conditions were all designated as Go trials, whereas the stop condition corresponded to No-Go trials.

Each trial sequence (Figure 1B) began with a central cross-shaped fixation point presented for 2000 ms, followed by the stimulus array. In Go trials, the participants responded by pressing different keys according to the direction of the central arrow (pressing “F” with the left hand for leftward arrows, or “J” with the right hand for rightward arrows). In No-Go trials, the participant were required to withhold their response. The response time window was 1300 ms. Response accuracy and reaction time (RT) were recorded for each trial. A correct keypress in a Go trial is recorded as a correct hit (CH); failure to respond in a Go trial is recorded as a miss (M). In No-Go trials, successful inhibition of a response (i.e., waiting for the stimulus to disappear automatically) is recorded as a correct rejection (CR); an incorrect keypress in a No-Go trial is recorded as a false alarm (FA). Based on the above metrics, post-error slowing (PES) and post-error accuracy (PEA) indices were further calculated to reflect the individual’s error monitoring abilities. Consistent with the previous literature [46], our analyses of post-error monitoring focused primarily on false alarms. Although both omissions and false alarms occurred in this task, they likely stem from distinct cognitive mechanisms. Omissions are typically attributed to delayed motor responses, whereas false alarms reflect failures in stimulus discrimination and inhibitory control, thereby involving greater top-down cognitive processing. Furthermore, given that false alarms constituted the predominant error type in this paradigm while omissions were rare, calculations were based on false alarms. The formulas for PES and PEA are as follows:

(1) $$\mathrm{PES}={\bar{\mathrm{RT}}}_{\mathrm{FA}\to \mathrm{CH}}-{\bar{\mathrm{RT}}}_{\mathrm{CH}\to \mathrm{CH}}$$

(2) $$\mathrm{PEA}=\frac{\sum \left(N_{\mathrm{FA}\to \mathrm{CH}}+N_{\mathrm{FA}\to \mathrm{CR}}\right)}{\sum \left(N_{\mathrm{FA}\to \mathrm{CH}}+N_{\mathrm{FA}\to \mathrm{CR}}+N_{\mathrm{FA}\to M}+N_{\mathrm{FA}\to \mathrm{FA}}\right)}$$

where ${\bar{\mathrm{RT}}}_{\mathrm{FA}\to \mathrm{CH}}$ denotes the average reaction time on correct hit trials immediately following a false alarm trial; ${\bar{\mathrm{RT}}}_{\mathrm{CH}\to \mathrm{CH}}$ denotes the average reaction time on the next correct hit trial following a correct hit trial; $N_{\mathrm{FA}\to \mathrm{CH}}$ denotes the number of trials in which a correct hit occurs immediately following a false alarm trial; $N_{\mathrm{FA}\to \mathrm{CR}}$ denotes the number of trials in which a correct rejection occurs immediately following a false alarm trial; $N_{\mathrm{FA}\to M}$ denotes the number of trials in which a false alarm is followed immediately by a miss; and $N_{\mathrm{FA}\to \mathrm{FA}}$ denotes the number of trials in which a false alarm is followed immediately by another false alarm.

### 2.4. Data Analysis

Data cleaning was performed prior to statistical analysis. Reaction times below 200 ms were excluded [47]. Additionally, data from an entire block were discarded if a participant exhibited more than 20 consecutive identical responses or non-responses within that block [48]. The remaining valid data were analyzed using SPSS 21.0 and RStudio 2024.04.0.

During the data analysis phase, linear mixed-effects models were fitted using the lme4 package in R to examine the effects of DD_status (with or without DD) and ADHD_status (with or without ADHD) on error monitoring performance, specifically the PES and PEA. The primary predictors included DD_status, ADHD_status, and their interaction. Sex, age, and non-verbal intelligence were included as covariates. To account for significant age differences between groups, interactions between age and diagnostic status (i.e., age × DD_status and age × ADHD_status) were also incorporated as fixed effects to control for potential confounding. A random intercept for the recruitment source was included to address the non-independence of observations across sites. Finally, hierarchical regression analyses were conducted to examine whether error monitoring independently predicted core symptom severity in dyslexia and ADHD. Z-scores in the character recognition tasks (representing reading ability), scores in the inattentive subscale, and scores in the hyperactive–impulsive subscale were regarded as dependent variables in the regression models. Covariates (i.e., age, sex, and non-verbal intelligence) were entered at the first step; PES and PEA were entered alternately at the second and third steps to assess their unique predictive contributions.

## 3. Results

### 3.1. Group Comparisons

**Figure 2:**
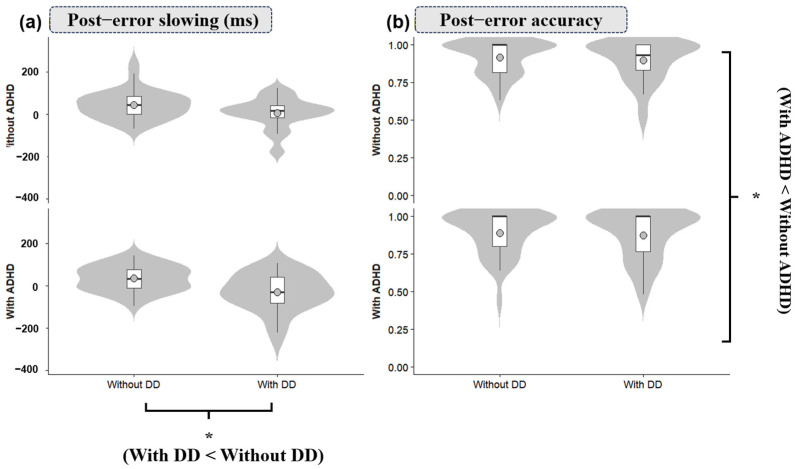
Comparison of post-error slowing (**a**) and post-error accuracy (**b**) across the four groups of children. Note: The figure above shows the main effects of DD_status and ADHD_status; * *p* < 0.05. The violin plots illustrate the distribution of the data, and the box plots indicate the medians and interquartile ranges.

The linear mixed-effects model with PES as the dependent variable and DD_status and ADHD_status as fixed factors (see Figure 2a) revealed a significant main effect of DD_status (*t* = 2.31, *p* = 0.02, *SE* = 82.90, *B* = −191.42, 95% *C.I.* [−349.91, −32.93]). Post-hoc comparisons indicated that children with DD showed significantly shorter PES than those without DD. The main effect of ADHD_status was not significant (*t* = 0.96, *p* = 0.34, *SE* = 118.46, *B* = 113.85, 95% *C.I.* [−112.62, 340.32]), and the DD_status × ADHD_status interaction was also not significant (*t* = 1.36, *p* = 0.18, *SE* = 30.28, *B* = −41.22, 95% *C.I.* [−99.11, 16.68]). A fixed effect was also found for sex (*t* = 2.31, *p* = 0.02, *SE* = 82.90, *B* = −191.42, 95% *C.I.* [−349.91, −32.93]), with males showing longer PES than females. No other fixed effects, including age-by-diagnosis interactions, reached statistical significance (*ps* > 0.1).

The linear mixed-models with PEA as the dependent variable (see Figure 2b) showed that the main effect of DD_status was not significant (*t* = 0.79, *p* = 0.43, *SE* = 0.16, *B* = 0.13, 95% *C.I.* [−0.19, 0.44]). The main effect of ADHD_status was significant (*t* = 2.18, *p* = 0.03, *SE* = 0.24, *B* = −0.51, 95% *C.I.* [−0.96, −0.06]). Post-hoc comparisons indicated that children with ADHD had lower PEA (M ± SD = 0.86 ± 0.15) than those without ADHD (M ± SD = 0.90 ± 0.12). The DD_status × ADHD_status interaction was not significant (*t* = 0.87, *p* = 0.39, *SE* = 0.06, *B* = 0.05, 95% *C.I.* [−0.06, 0.17]). Neither the effects of sex nor age were significant (*ps* > 0.1), whereas the covariate effect of non-verbal intelligence was significant (*t* = 3.38, *p* < 0.001, *SE* < 0.001, *B* = 0.003, 95% *C.I.* [0.001, 0.004]), indicating a trend that higher non-verbal intelligence was associated with higher PEA. The age-by-diagnosis interactions were not significant (age × DD_status: *t* = 0.91, *p* = 0.36, *SE* = 0.02, *B* = −0.02, 95% *C.I.* [−0.06, 0.02]; age × ADHD_status: *t* = 1.61, *p* = 0.11, *SE* = 0.03, *B* = 0.05, 95% *C.I.* [−0.01, 0.10]). Together with the PES findings, these results indicates that age did not significantly moderate the impact of DD_status or ADHD_status on error monitoring.

The random intercept variances in the linear mixed-effects model were significant for PEA (*t* = 2.81, *p* = 0.006, *SE* = 0.24, *B* = 0.67, 95% *C.I.* [0.21, 1.23]) instead of PES (*p* > 0.1), indicating significant differences between recruitment sources and validating the suitability of the mixed model. Detailed information regarding the error monitoring function of each group is shown in Table 2.

### 3.2. Hierarchical Regression Analyses

In the hierarchical regression models, Z-scores in the character recognition tests (representing Chinese-character reading ability), scores in the inattentive subscale, and scores in the hyperactive–impulsive subscale were separately used as dependent variables. Age, sex, non-verbal intelligence (i.e., testing scores in the RSPM test), and DD/ADHD status were firstly entered into the model, while the second and third steps alternately included PES and PEA, respectively. As shown in Table 3, the results indicated that only PES, but not PEA, independently and significantly explains 2% of the variance in Chinese-character reading (*β* = 0.12, *t* = 2.03, *p* = 0.04, 95% *C.I.* [<0.001, 0.003]); neither of the two error monitoring indices had a significant predictive effect on inattention or hyperactivity–impulsivity (*ps* > 0.1).

## 4. Discussion

This study examined error monitoring across four groups of children (i.e., TD, DD-only, ADHD-only, and comorbid) using two behavioral indices of PES and PEA. Results from the linear mixed-effects models revealed that children with DD (including both the comorbid and DD-only groups) exhibited significantly shorter PES than children without DD (including the ADHD-only group and the TD group), with no significant difference observed between the DD-only and comorbid groups. Conversely, children with ADHD (including both the ADHD-only and comorbid groups) exhibited lower PEA than those without ADHD (DD-only and TD groups), and PEA levels were comparable between the ADHD-only and comorbid groups. These findings suggest distinct alterations in error monitoring between DD and ADHD. Particularly, children with DD primarily manifest difficulties in error detection, whereas those with ADHD exhibit weak error regulation. Furthermore, the results from hierarchical regression analyses indicated that PES significantly and positively predicted reading abilities but did not significantly predict ADHD symptoms (including inattention or hyperactivity–impulsivity). PEA showed no significant predictive effect on either reading ability or ADHD symptoms, suggesting an association between error detection and reading acquisition.

This study found that children with DD (both the comorbid and DD-only groups) exhibited a significantly shorter PES than those without DD, which aligns with previous findings [19,20,21,22] and our expectation, indicating that error monitoring functions (particularly the subcomponent of error detection) are atypical in children with DD. The shortened PES suggests that children with DD may fail to effectively detect errors, consequently lacking the impetus for such compensatory slowing [19]. To dissect this deficit temporally, several studies have employed the event-related potential (ERP) technique to dissociate the different processing stages of error detection [49,50]. Specifically, the Error-Related Negativity (ERN), occurring 0–160 ms post-error, reflects rapid, automated, and unconscious conflict monitoring; whereas the Error Positivity (Pe), occurring 200–500 ms post-error, is associated with conscious error awareness and evaluation [51,52]. Previous research indicates that children with DD exhibit significantly reduced amplitudes for both ERN and Pe compared to TD children, suggesting that their alterations in error detection manifest at multiple stages, including an inability to rapidly and automatically detect errors at early stages, coupled with difficulties in consciously evaluating errors at later stages [49,50,53,54].

Furthermore, our findings reveal that PES significantly predicts children’s reading ability, suggesting an association between error monitoring and Chinese character reading. From a cognitive perspective, reading is a dynamic process reliant on real-time self-monitoring, where proficient readers initiate adaptive adjustments immediately upon detecting a conflict or error [54]. Specifically, early, automated error detection unconsciously compares visual input with internal phonological representations within milliseconds, rapidly intercepting orthography–phonology conflicts to ensure fluent word recognition. Conversely, late, conscious error evaluation redirects attentional resources to the erroneous stimulus, prompting in-depth orthographic analysis and strategic corrections, thereby solidifying correct orthography–phonology mappings in long-term memory. Weak error detection prevents children with dyslexia from timely suppressing incorrect reading impulses or mobilizing resources for deep processing when encountering orthography–phonology conflicts [55]. This may hinder the establishment of stable character–sound correspondences, limiting their character recognition and literacy acquisition to some extent. From a neurobiological perspective, the association between error detection and reading may stem from the functional specificity of the anterior cingulate cortex (ACC) and its associated networks. The ACC serves not only as a monitoring hub that generates ERN signals and triggers behavioral adjustments [56], but also as a key node in processing orthography–phonology conflicts during reading [57]. Children with DD have been observed to exhibit delayed structural development and functional abnormalities of the ACC, along with altered functional connectivity with the dorsolateral prefrontal cortex and insula [58,59,60]. These abnormalities may constitute a shared neural basis for both poor error monitoring and reduced reading efficiency in DD.

This study found no significant differences in PES scores between children with ADHD and those without ADHD, which contradicts our expectation and some previous research [30,32]. Previous research suggests that PES performance in ADHD may be modulated by factors such as the task cognitive load and the inter-trial interval (ITI); specifically, under conditions of low cognitive load and longer ITI, PES differences between individuals with ADHD and typical peers often diminish [35]. However, the current study employed a task with a higher cognitive load than previous paradigms. Whereas prior studies required only single-dimensional button-pressing, the present task required participants to determine whether to respond based on peripheral stimuli while simultaneously performing selective button-pressing according to the direction of a central arrow. Furthermore, while previous studies utilized an ITI of approximately 4–5 s, the interval in this study was shorter (approximately 3 s). Given that higher cognitive demands and shorter intervals typically exacerbate task difficulties, the comparable PES levels observed between children with ADHD and those without ADHD in this study are unlikely to be attributable to these methodological factors.

Alternatively, insights from ERP studies offer a plausible explanation. Research has shown that children with ADHD exhibit ERN abnormalities, reflecting impaired early, unconscious error detection [61]. However, the behavioral measure of PES used in this study reflects the macroscopic outcome of the transition from error detection to behavioral adjustment; it may lack the sensitivity to capture the nuances of purely early, automatic error processing. It is plausible that while unconscious error detection is impaired in the early stages for children with ADHD, they may compensate by maintaining post-error reaction time adjustments at the behavioral level through strategies employed in later processing stages. Therefore, future research urgently needs to combine ERP techniques with high temporal resolution to dissociate the distinct stages of post-error processing. This approach would clarify the nature of error monitoring in children with ADHD and investigate whether early unconscious error detection serves as a transdiagnostic risk factor for both DD and ADHD, or whether it is specific to DD.

This study found that children with ADHD exhibited lower PEA than their non-ADHD counterparts, indicating a potential association between ADHD and atypical error regulation. This result aligns with our prediction and previous research demonstrating that children with ADHD show a weaker PEA effect compared to typically developing children [33,50]. Conversely, no significant differences in PEA were observed between children with and without DD, suggesting that error regulation functions in children with DD remain relatively intact. This finding corroborates recent research by Zhai et al. [19], which revealed that deficits in children with DD are primarily manifested under unaware error conditions; under aware error conditions, however, their saccadic error rates do not differ significantly from those of age-matched typically developing children, indicating that the core issue in DD lies in unaware error detection rather than subsequent adjustment. From a cognitive mechanism perspective, PEA reflects an individual’s dynamic allocation of cognitive control resources following error detection. This process falls within the domain of post-error adjustment and is highly dependent on top-down executive control functions [13]. The core pathological mechanism of ADHD involves deficits in the executive control network, particularly during the post-error adjustment phase. This often manifests as insufficient activation of cognitive control-related brain regions (e.g., the dorsolateral prefrontal cortex) or abnormal ERP components [62], making it difficult for individuals to effectively translate error signals into behavioral adjustment strategies, thereby resulting in reduced or absent PEA. In contrast, executive control functions in DD are relatively preserved [63]. Consequently, error regulation (i.e., a process demanding robust cognitive control and attentional resources) more specifically reflects the core impairment in ADHD, making PEA abnormalities more closely associated with ADHD than with DD.

In the current study, children in the comorbid group exhibited a combination of the error monitoring alterations observed in the single disorders: a shortened PES similar to the DD-only group, alongside a reduced PEA comparable to the ADHD-only group. This suggests that comorbidity involves the concurrence of difficulties in both error detection and regulation, which was consistent with our expectation. However, the absence of a significant DD_status × ADHD_status interaction indicates that this dual alteration is statistically additive of the single-disorder problems, rather than representing a qualitatively distinct mechanism. While this pattern may be conceptually related to the stage-specific processing hypothesis proposed by Zhou et al. [64], our current data do not support a qualitatively distinct mechanism for comorbidity. Instead, the findings highlight the cumulative cognitive burden of having both conditions.

Nevertheless, the present study still has several limitations. First, our assessment of reading ability relied solely on character recognition and ADHD symptoms were parent-reported. Future research should incorporate multidimensional reading measures (e.g., word/text-level reading accuracy and passage comprehension) to provide a more comprehensive profile of reading abilities. Moreover, it should be noted that different screening tools of reading abilities were used across recruitment sites. Although Z-score normalization was applied to harmonize the data, the group-administered character recognition and assessment scale involves written responses (forming compound words or Pinyin annotation). Consequently, performance on this measure may be potentially confounded by handwriting fluency and vocabulary size, distinct from pure character recognition. Although there were no significant differences in the error monitoring functions between the two types of recruitment methods within each DD subgroup (see Table S2 in Supplementary Materials), future studies should consider using identical assessment tools across all sites to ensure strict psychometric equivalence. Furthermore, ADHD symptom levels were evaluated exclusively via parent-reported scales. Future studies could employ objective metrics, such as reaction time variability from continuous performance tests, to provide a more robust assessment of ADHD symptoms. Second, the medication status of children with ADHD was not controlled. Previous research indicates that stimulant medications can enhance error monitoring functions [40]. As the participants with ADHD were primarily recruited from clinical hospitals, some were likely undergoing medication, which may partially explain why this study did not find a greater severity of error monitoring problems in the ADHD groups. Therefore, the results pertaining to error monitoring in ADHD participants should be interpreted with caution. Future research should strictly record and control for medication status. Third, while we calculated post-error monitoring indices based on false alarms following established protocols, the task difficulty in the current study was relatively low, yielding an average of 14–16 false alarms per group. This limited number of valid post-error sequences likely contributed to increased data variability. Future research should aim to adjust task parameters to elicit more errors, ensuring a more robust and valid measurement of post-error monitoring. Moreover, it is important to acknowledge the limitations associated with the online administration of the error monitoring task via PsyToolkit. While web-based platforms offer scalability, they inherently lack the strict environmental control and hardware standardization of laboratory settings. Potential sources of variability include differences in participants’ display refresh rates, input device latencies (e.g., keyboard vs. laptop keys), and uncontrolled testing environments (e.g., background noise or distractions). These factors are particularly consequential for reaction-time-based measures like PES, which rely on precise millisecond-level differences between post-error and post-correct trials. The observed variability in our data, especially exemplified by the wide confidence intervals for the DD/ADHD_status effects on PES, may partly reflect this measurement noise introduced by the online delivery format. Therefore, the results regarding PES should be interpreted with caution, and future studies should aim to replicate these findings using controlled in-lab settings with high-precision timing equipment. Furthermore, relying solely on behavioral data limits the ability to distinguish between early automatic error detection and later conscious error awareness. Future studies should combine high-temporal-resolution techniques, such as ERP and eye-tracking, to further dissect the neural temporal characteristics across different sub-processes of error monitoring in children with DD and ADHD. Finally, this study employed a cross-sectional design, which precludes establishing the directionality of the relationship between error monitoring and DD/ADHD symptoms. Subsequent research should adopt longitudinal designs or intervention-based paradigms to further explore the developmental influence of error monitoring on these disorders and to clarify potential causal relationships.

## 5. Conclusions

Based on a combined Flanker–No-Go task utilizing two key post-error indicators (i.e., PES and PEA), this study systematically compared error monitoring functions across four groups of children, including those with DD only, ADHD only, comorbid DD + ADHD, and their TD peers. The results revealed that children with DD (including both the comorbid and DD-only groups) exhibited significantly shorter PES compared to their non-DD counterparts. Notably, the PES scores of the comorbid group were comparable to those of the DD-only group, suggesting that children with DD experience significant difficulties in error monitoring, particularly in error detection. Furthermore, PES scores were found to predict character recognition to some extent. Simultaneously, children with ADHD (including both the ADHD-only and comorbid groups) demonstrated lower PEA scores than those without ADHD, indicating that error regulation functions may be atypical in children with ADHD. These findings suggest that while atypical error monitoring represents a shared characteristic in both DD and ADHD, the specific manifestations differ between the two disorders: DD is closely associated with altered error detection, whereas ADHD is possibly linked to diminished post-error correction abilities. These findings tentatively suggest that future interventions for dyslexia could incorporate error detection sensitivity training as a supplementary component to potentially enhance overall efficacy. Similarly, for children with ADHD, interventions might prioritize strengthening post-error correction functions. For comorbid cases, it is hypothesized that comprehensive programs addressing both error detection and regulation mechanisms would be most beneficial, though these specific intervention effects remain to be empirically validated.

## Figures and Tables

**Table 1:** Descriptive statistics and group comparison results across the four groups.

	TD Group ① (*n* = 34)	DD Group ② (*n* = 37)	ADHD Group ③ (*n* = 37)	Comorbid Group ④ (*n* = 40)	*F*	*η*^2^ 95% *C.I.*	Post-Hoc Comparisons
Recruitment sources							
Hospital recommendation	0	1	21	21	—	—	—
Online advertisement	2	2	9	2	—	—	—
Elementary school 1	20	20	5	8	—	—	—
Elementary school 2	12	14	2	9	—	—	—
Age (years)	7.93 (0.53)	8.05 (0.69)	9.98 (1.28)	10.02 (1.63)	37.76 ***	0.44 [0.32, 0.53]	③④ > ①②
Inattention	12.41 (8.31)	11.59 (7.05)	17.95 (4.74)	19.17 (5.35)	13.22 ***	0.22, [0.10, 0.32]	③④ > ①②
Hyperactivity–impulsivity	7.68 (7.72)	4.94 (6.78)	11.86 (6.48)	11.58 (5.97)	9.11 ***	0.16 [0.06, 0.26]	③④ > ①②
Vocabulary (Z-score)	0.70 (0.68)	−0.82 (0.85)	0.55 (0.69)	−0.43 (0.99)	29.88 ***	0.38 [0.26, 0.48]	①③ > ④ > ②
RSPM (percentile rank)	81.67 (13.79)	81.42 (11.68)	86.69 (15.25)	82.18 (12.83)	1.26	—	—
EM accuracy	90.09% (7%)	89.54% (7%)	89.70% (9%)	89.78% (12%)	0.02	—	—

Note: TD, typically developing children; DD, dyslexia; ADHD, attention deficit hyperactivity disorder. Because two different vocabulary measures were used to identify individuals with developmental dyslexia in this study, scores from each measure were normalized to Z-scores for group comparisons. RSPM, the percentile rank of the testing score in the Raven’s standard progressive matrices test. EM accuracy, the mean accuracy in the error monitoring task. *** *p* < 0.001.

**Table 2:** Descriptive statistics information of error monitoring functions for the four groups.

	PES (ms)		PEA	
	*M (SD)*	Range	*M (SD)*	Range
Four groups				
TD	53.44 (58.89)	−43.34–241.64	0.91 (0.11)	0.63–1
DD	−18.25 (68.68)	−187.22–112.16	0.89 (0.15)	0.5–1
ADHD	41.91 (54.70)	−55.02–142.46	0.87 (0.15)	0.44–1
Comorbid	−41.55 (85.14)	−269.42–99.25	0.85 (0.16)	0.48–1
DD_status				
With DD	−30.36 (78.04)	−269.42–112.16	0.87 (0.15)	0.48–1
Without DD	47.43 (56.63)	−55.02–241.64	0.89 (0.13)	0.44–1
ADHD_status				
With ADHD	−1.44 (83.06)	−269.42–142.46	0.86 (0.15)	0.44–1
Without ADHD	16.08 (73.22)	−187.22–241.64	0.90 (0.12)	0.5–1

Note: TD, typically developing children; DD, dyslexia; ADHD, attention deficit hyperactivity disorder; comorbid, children with the comorbidity of dyslexia and ADHD. PES, post-error slowing; PEA, post-error accuracy.

**Table 3:** Results of hierarchical regression analyses.

Block	Variable	Chinese Character Reading				Inattention				Hyperactivity-Impulsivity			
		*β*	*t*	*Δ* *R* ^2^	*ΔF*	*β*	*t*	*Δ* *R* ^2^	*ΔF*	*β*	*t*	*Δ* *R* ^2^	*ΔF*
Block 1				0.54	32.77 ***			0.23	8.54 ***			0.17	5.73 ***
	Age	0.49	6.21 ***			0.08	0.83			0.02	0.16		
	Sex	−0.05	0.94			−0.05	0.68			−0.10	−1.30		
	RSPM	0.00	0.06			−0.10	1.35			−0.10	−1.20		
	ADHD_status	−0.26	3.26 **			0.39	3.73 ***			0.36	3.28 **		
	DD_status	−0.59	9.78 ***			0.03	0.42			−0.05	−0.60		
Block 2				0.02	4.20 *			0.004	0.66			0.01	2.70
	PES	**0.12**	2.03 *			0.07	0.92			0.13	1.61		
Block 3				<0.001	0.003			0.005	0.85			<0.001	0.01
	PEA	−0.003	0.05			−0.07	0.92			0.01	0.11		
Block2				<0.001	0.04			0.004	0.67			<0.001	0.09
	PEA	−0.003	0.05			−0.07	0.92			0.01	0.11		
Block3				0.02	4.14 *			0.005	0.85			0.01	2.60
	PES	**0.12**	2.03 *			0.07	0.92			0.10	1.61		

Note: RSPM, the percentile rank of the testing score in the Raven’s standard progressive matrices test. PES, post-error slowing; PEA, post-error accuracy. The significant predictions are marked in bold. * *p* < 0.05, ** *p* < 0.01, *** *p* < 0.001.

## Data Availability

To protect the privacy of participants, the raw data will not be publicly available. However, the data are available upon reasonable request and with the approval of the School of Psychology, Capital Normal University. Researchers interested in accessing the data may submit an application to the corresponding author via email at zhaojing@cnu.edu.cn.

## Supplementary Materials

The following supporting information can be downloaded at https://www.mdpi.com/article/10.3390/brainsci16070669/s1. Table S1: group comparisons between two versions of error monitoring tasks. Table S2: DD subgroup comparisons in error monitoring between two types of recruitment methods.

## Abbreviations

The following abbreviations are used in this manuscript:

DD	Developmental dyslexia
ADHD	Attention deficit hyperactivity disorder
TD	Typically developing
PES	Post-error slowing
PEA	Post-error accuracy
ERN	Error-Related Negativity
Pe	Error Positivity
