# Supplementary material for: Dissociable Patterns of Atypical Error Monitoring in Developmental Dyslexia and Attention Deficit Hyperactivity Disorder
Source: Brain Sci. 2026 Jun 26;16(7):669. doi: 10.3390/brainsci16070669 (PMC13406497; doi:10.3390/brainsci16070669)
Supplement: Supplementary file 1 [file brainsci-16-00669-s001.zip › brainsci-4352913-supplementary.pdf]

Supplementary Materials

Table S1 Group comparisons between two versions of error monitoring tasks

	**Three-version**	**Four-version**	**t values**	**p values**	**95% *C.I.***
**PES** (ms)	-76.13(97.33)	-31.51(80.21)	1.40	0.17	[-109.09, 19.84]
**PEA**	0.86(0.17)	0.85(0.16)	0.30	0.77	[-0.11, 0.14]

Note. PES, post-error slowing; PEA, post-error accuracy. Datasets were all from children with the comorbidity of dyslexia and ADHD.

Table S2 DD subgroup comparisons in error monitoring between two types of recruitment methods

	**Hospital+Online**	**Elementary school**	**t values**	**p values**	**95% *C.I.***
Within the DD-only group					
**PES** (ms)	-5.10(94.12)	-19.41(67.76)	0.34	0.74	[-70.71, 99.33]
**PEA**	0.88(0.04)	0.90(0.14)	0.15	0.88	[-0.18, 0.15]
Within Comorbid group					
**PES** (ms)	-36.04(82.08)	-49.00(91.11)	0.47	0.64	[-42.73, 68.64]
**PEA**	0.88(0.17)	0.81(0.15)	1.27	0.21	[-0.04, 0.17]

Note. PES, post-error slowing; PEA, post-error accuracy.
